# Exploring factors influencing integration of traditional and medical male circumcision methods at Ingquza Hill Local Municipality, Eastern Cape: A socio-ecological perspective

**DOI:** 10.4102/phcfm.v11i1.1948

**Published:** 2019-08-06

**Authors:** Sandile Prusente, Nelisiwe Khuzwayo, Yandisa Sikweyiya

**Affiliations:** 1 School of Nursing and Public Health, University of KwaZulu-Natal, Durban, South Africa; 2 Gender and Health Research Unit, South African Medical Research Council, Pretoria, South Africa; 3 School of Public Health, University of the Witwatersrand, Johannesburg, South Africa

**Keywords:** male circumcision, social acceptance, discrimination, health, culture

## Abstract

**Background:**

Medical male circumcision (MMC) and traditional male circumcision (TMC) are reportedly having negative and positive outcomes in the Eastern Cape province. Researchers show contradictory remedies; some advocate for abolishment of TMC and others call for the integration of both methods.

**Aim:**

This study aimed to explore factors influencing the integration of TMC and MMC at different socio-ecological levels.

**Setting:**

The study was conducted at Ingquza Hill Local Municipality in the Eastern Cape province.

**Methods:**

An explorative qualitative study design, using in-depth interviews (IDIs) and focus group discussions (FGDs), was employed in this study. Purposive sampling was used to select the participants. A framework analysis approach was used to analyse the data, and the themes were developed in line with the socio-ecological model.

**Results:**

Four main themes emerged from the data as important in influencing the integration of TMC and MMC methods. These included: (1) individual factors, related to circumcision age eligibility and post-circumcision behaviour; (2) microsystem factors, related to alcohol and drug abuse, peer pressure, abuse of initiates, and family influence; (3) exosystem factors, related to financial gains associated with circumcision and the role of community forums; and (4) macrosystem factors, related to stigma and discrimination, and male youth dominance in circumcision practices.

**Conclusion:**

Male circumcision in this area is influenced by complex factors at multiple social levels. Interventions directed at all of these levels are urgently needed to facilitate integration of the TMC and MMC methods.

## Introduction

In South Africa, male circumcision (MC) is one of the oldest traditions practised by various black African ethnic groups. It involves the surgical removal of all or part of the foreskin of the penis. In numerous African settings, MC plays an integral role in the identity development of young men as it is viewed as a rite of passage from boyhood to manhood^1^; however, it is also performed for religious and medical reasons.^2,3^ Traditional male circumcision (TMC) is performed in a traditional setting by a traditional surgeon (ingcibi) who has undergone the same procedure using a traditional spear; a traditional nurse (ikhankatha) is then tasked with ensuring safety and care of the initiates and monitoring the healing process while constantly offering teachings related to manhood.^3^ Generally, boys would be taken away from their homes with or without the permission of the head of the families and kept in a secluded place where they will be circumcised and kept for a period of 2 to 4 weeks to allow the healing process. TMC is principally practised by Southern and Northern Nguni ethnic groups as a catalyst that informs the self-identity of African youth.^4^ TMC falls under the legal jurisdiction of traditional leadership, which acts as custodian of the custom and cultural practices. Studies^3^ have found that men in the Eastern Cape province largely prefer TMC over medical male circumcision (MMC), although there is no strong scientific evidence showing its health benefits compared to MMC.

Medical male circumcision is performed in a clinic or hospital setting by a trained and competent health practitioner using surgically clean material, blades and sutures, and the procedure is performed under local anaesthesia while observing for adverse symptoms. MMC has been identified as one of the key prevention strategies for reducing the risk of HIV acquisition through female-to-male transmission during sexual intercourse. Three ground-breaking trials conducted in various African countries have shown that MMC reduces female-to-male transmission of HIV by up to 60%.^5,6^ As MMC was found to be effective in reducing HIV,^7^ many African countries, including South Africa, are offering the service freely to all males, especially those in non-circumcising communities. Although MMC has been associated with health benefits in South Africa, its uptake has not been as high as the government had hoped.^8^ From April 2015 to March 2016, 464 731 male circumcisions had been performed.^9^ However, authors^10^ argue that MMC is not generally accepted, especially among ethnic groups that practise TMC.

The two male circumcision methods differ in age eligibility criteria, with the former set at 18 years and the latter has no age limitation in South Africa. A study conducted in the Eastern Cape^2^ revealed that Xhosa initiates reported mixed attitudes towards combining MMC and TMC, with the majority feeling that they could be stigmatised for selecting MMC over TMC.^2^ Presently in South Africa, the custodians of culture are searching for solutions to the serious health-related problems faced by TMC, instead of considering MMC as an alternative.^11^ Statistics from the Commission for the Promotion and Protection of the Rights of Cultural, Religious and Linguistic (CRL) Communities show that 774 initiates died across eight provinces in South Africa between 2006 and 2016. It is further reported that between 2014 and 2016, 199 initiates died in the Eastern Cape,^12,13^ and that most of these deaths were in OR Tambo District Municipality. In response, the South African government has recognised TMC-related morbidity and mortality as a serious public health problem that needs collaborative intervention between the traditional leadership (as the custodians of the custom), communities, families, and relevant government authorities.^14^

Review of the existing MC policies in order to enable the integration of MMC and TMC is urgently required. Owing to the continuing poor health and social outcomes in TMC, this study aimed to explore socio-ecological factors influencing the integration of traditional and medical male circumcision at the Ingquza Hill Local Municipality. It is common that TMC in South Africa is faced with challenges, including death and the abuse of initiates. While MMC is presumed to be safe, initiates and men who have been circumcised through this method are often confronted with social exclusion and discrimination. Integration in this context refers to the merger of the two male circumcision methods where positive practices from both methods can be synergised to improve health and social outcomes (e.g. reduction of deaths from TMC and infections) of the service users without denigrating any method.

### Theoretical framework

In the Eastern Cape province, MMC and TMC are a public concern and thus researchers call for the integration of these methods.^2^ Both methods have been reported to provide benefits, either medically or socially. Ecological theory was deemed appropriate by the researchers in exploring factors that can influence integration of these methods. Ecological theory provides a comprehensive picture of a phenomenon under investigation as it provides a multifaceted understanding and explains interactive relationships between people and their environments.^15,16^ The theory contains four levels, which are described in the following:

- Intrapersonal level (microsystem): this level includes family, peers, school, church, health services and neighbourhood with which an individual regularly interacts; it is the most powerful level of influence.
- Interpersonal level (mesosystem): this level involves interactions between people who are in the first level, where these interconnections between them will have an indirect impact on the individual.
- Community level (exosystem): an individual is not an active participant in this level, but is indirectly affected by all activities within the community. At this level, decision-making authorities make binding decisions on behalf of individuals without their participation and these require compliance.
- Societal level (macrosystem): this level consists of broad factors such as cultural values, customs, beliefs, traditions and laws. Social and gender inequality, masculinity (also linked to dominance) and social and cultural norms have a cascading influence throughout the interactions of all other levels.

## Methods

### Study design

An explorative qualitative approach was employed to explore factors influencing the integration of traditional and medical male circumcision methods at the Ingquza Hill Local Municipality, Eastern Cape province.

### Study setting

The study was conducted at the Ingquza Hill Local Municipality, specifically in the rural communities of Lusikisiki and Flagstaff, in the Eastern Cape province. This municipality was purposively selected because of its very high mortality rate among traditionally circumcised initiates in the Eastern Cape province. It has a total population of 303 379^15^ and is predominantly constituted of black Africans who belong to the AmaMpondo ethnic group. A low adult literacy rate and high levels of poverty characterise it.^17^

### Study population and sampling

The study population comprised a diverse group of community men aged 20–79 years who were either traditionally or medically circumcised, or uncircumcised. Also, a diverse group of community women aged 25–59 years formed a part of the study sample. Both groups comprised female and male traditional leaders, traditional surgeons and healthcare workers based in public health facilities at the Ingquza Hill Local Municipality who were interviewed in this study. All participants spoke isiXhosa, which is the dominant language spoken in the area. Tables 1 and 2 present the characteristics of the participants.

**TABLE 1:** Socio-demographic characteristics of focus group discussion participants.

FGD	Age (year)	Gender	Role in the community	Number of respondents
FGD 1	55–65	Males	Members of the council	7
FGD 2	20–35	Males	Community members	6
FGD 3	25–49	Females	Community member	7
FGD 4	20–35	Males	Community members	6

FGD, focus group discussion.

**TABLE 2:** Socio-demographic characteristics of in-depth interview participants.

Age (year)	Gender	Role in the community	Number of respondents
30–59	Male (2), Female (1)	Healthcare worker	3
20–59	Male	Community member	3
30–35	Male	Traditional Circumcision Forum	1
45–55	Male	Traditional surgeon	2
45–79	Male (5), Female (1)	Local Traditional leader	6
35–40	Male	Traditional nurse	1

IDI, in-depth interviews.

A purposive sampling method was used to select the participants. The first author (S.P.) engaged local traditional councils to request permission to conduct the study in the area; permission was granted after approval had been given by the House of Traditional Leaders based in Bhisho, Eastern Cape province. The first author initiated consultations with local traditional councils where he gave brief presentations of the study to the heads of the councils who then advised him to present the study in their weekly council meetings. The first author then attended weekly council meetings where he presented the aim of the study, procedures involved, study duration, proposed participants and how their identity will be protected, and other safety and ethical measures that will be implemented to ensure that the study was conducted in a safe and ethical manner. After careful consideration by the Head of the Council and traditional council members, a letter granting permission to conduct the study was issued. The names of local chiefs were obtained from the local traditional council database. Local traditional leaders were then asked to help with the recruitment of participants as they were conversant with the geographical distribution of communities that fall under their authority, and with cultural and religious practices in their respective communities. Other participants were recruited through facility managers and the male circumcision forum operating in this municipality. Traditional leaders were the most trusted figures in these communities; they did not influence the first author on who exactly must be selected to participate in the study.

### Data collection

The data were derived from focus group discussions (FGDs) and in-depth interviews (IDIs), which broadly explored socio-ecological factors influencing the integration of TMC and MMC methods. A total of 42 participants participated in the study. A total of four FGDs were conducted with 26 participants who did not take part in the IDIs: one with elderly men, two with each group of young men and one with women of diverse ages. Each FGD had between six and seven participants and they were all facilitated by the first author. The discussions included exploring reasons for choosing a particular circumcision method over another, knowledge and understanding of MMC and TMC, experience of undergoing the chosen method (for circumcised males), thoughts and opinions about MMC and TMC, and challenges facing MMC in the area. The FGDs lasted between 1 h and 2 h.

With the use of an interview guide, IDIs were conducted with at least 16 participants in a private space to enhance the confidentiality of participants’ information and to explore in detail the respondents’ own perceptions and accounts without any disturbances from other people. Participants for the IDIs included elderly men, female and male traditional leaders, traditional surgeons, nurses and other healthcare workers. The discussions included exploring reasons for choosing a particular circumcision method over another, knowledge and understanding of the MMC and TMC, experience of undergoing the chosen method (for those circumcised), thoughts and opinions about MMC and TMC, and challenges facing MMC in the area. Audio-recorded IDIs and FGDs were conducted in isiXhosa by a male researcher (S.P.). IsiXhosa is the most frequently spoken language in the study setting. IDIs lasted between 30 min and 1 h.

**FIGURE 1:**
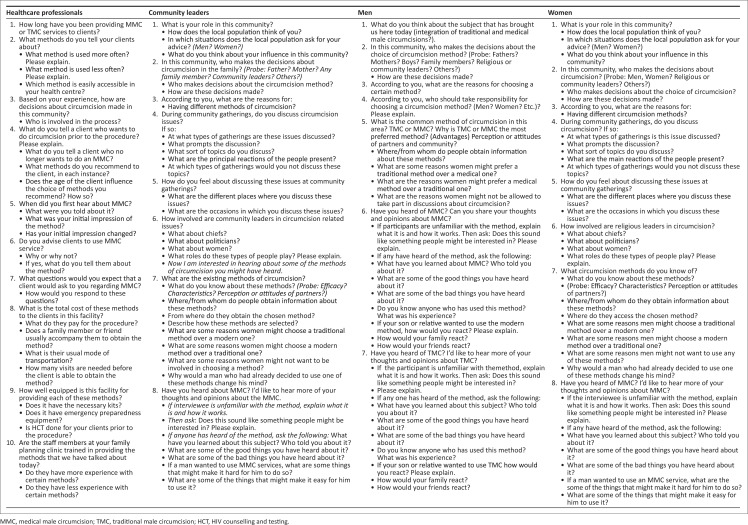

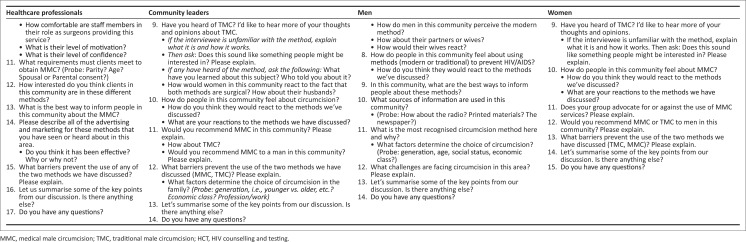
Interview guides.

Data collection was conducted from August to November 2015, and Figure 1 presents interview guides for respondents. In preparation for data analysis, transcripts from both IDIs and FGDs were translated into English by the first author and checked for accuracy by the other authors. The first author stopped data collection after realising that there was no new information being shared by the participants. From the 10th interview, no new information was forthcoming and this convinced us that saturation had been reached. However, in order to ascertain this, a few more interviews were conducted. At interview 16, we were convinced that saturation had been reached and IDIs were discontinued.

### Data analysis

Data were analysed following the framework analysis approach. The authors felt that this was the most suitable method as it emphasises how both *a priori* issues and emergent themes could guide the development of the analytic framework.^17^ This fitted the aim of the study as the authors had certain predefined areas to explore but also wanted to remain open to discovering the unexpected. Using the five stages of framework analysis, the first author listened to the audio recordings of the IDIs and FGDs and transcribed them. All authors then read the transcripts and deliberated on emerging issues in the data; in this process, the authors aimed at *familiarising* themselves with the data. All of the authors conducted *coding* separately. To ensure inter-rater consistency, the authors compared and discussed the coding of transcripts. Then similar codes were grouped to form the categories (*indexing*). Bronfenbrenner’s (1996) social-ecological approach was identified as a useful framework for the purposes of sifting and sorting data, rather than thinking about the data set in a more interpretative way. The transcripts were then organised into the framework category, systematically applying the framework to each transcript.

*Indexed* data for each category were summarised into a *chart form*; at this stage, the aim was to condense the data set into a more manageable form. Having summarised all the data from the IDIs and FGDs in this way, the authors moved to the final stage of the framework analysis. This involved finding patterns, pulling together key characteristics of the data to map and interpret the data set as a whole, which included description, clarification of concepts and representing the range and the nature of phenomena within the data. Validity in this study was ensured through consistency of findings, and adequate and systematic use of the original data, for example, using quotations from various IDIs and FGDs.

### Reflexivity

We consistently observed reflexivity through emphasis on objectivity and detachment, and suspension of biasness and preconceived ideas.^18^ The authors originate from an African context and are familiar with both male circumcision methods; furthermore, they were fully cognisant and objective in the interpretation of data without inadvertently imposing their personal beliefs and assumptions on the data. A ‘decision trail’ was constructed, explaining the selection criteria of participants as clearly articulated in the methods section, and development of the questions assisted in addressing reflexivity. The authors interrogated the purported problem, examining its veracity, how it is constituted, what it means for those involved – parents, traditional leaders, youth and everyone affected by this problem – and how it can be successfully resolved. Careful consideration was observed from the onset of the study, participants were informed about the nature of the study and their anonymity was assured and pseudonyms were used in reporting the findings. The participants were prompted, probed and encouraged to express their views and experiences in order to develop interactive relationships and solicit first-hand experience providing valuable and meaningful data. The data were recorded and transcribed by the first author to gain an awareness of gaps and subtle ways that may have led to biased data collection. The data were then re-interpreted and re-gathered in areas that were outside the first author’s initial assumptions and contained enlightening revelations.

### Ethical considerations

Ethical approval for this study was obtained from the Biomedical Research Ethics Committee (BREC) (Ref No.: BE 175/15) of the University of KwaZulu-Natal. Permission was obtained from the Eastern Cape Department of Health (Ref No.: EC_2015RP4_3). A traditional leader who was a member of the Eastern Cape House of Traditional Leaders also gave permission. Written informed consent was obtained from all the participants prior to their inclusion in the study. All the participants who participated in the FGDs were reimbursed for their transport costs and a finger lunch was provided for them.

## Results

Data in this study revealed that circumcision behaviour was influenced by individual and contextual factors at different levels. These included (1) individual factors (circumcision age eligibility and post-circumcision behaviour), (2) microsystem factors (alcohol and drug abuse, initiate abuse and family influence), (3) exosystem factors (circumcision and financial gains and the role of community forums) and (4) macrosystem factors (stigma and discrimination and the dominance of young men in the TMC process) as key factors influencing the integration of traditional and medical male circumcision methods in the setting of this study.

### Individual factors

As shown below, post-circumcision behaviour and circumcision age eligibility emerged as key individual factors that undermined the integration of TMC and MMC circumcision methods in this setting. These are reported below.

#### Post-circumcision behaviour

Data in this study suggest that most young men change their behaviour after undergoing circumcision. Yet, this change in behaviour was often undesirable and at odds with the behaviour expected of a man who has undergone circumcision in this setting. This undesirable post-circumcision behaviour, often displayed by newly circumcised men (*amakrwala*) who are traditionally circumcised, was directly linked to the teachings offered in the initiation school. In the following extract, a headman laments the undesirable behaviour of the newly circumcised. He explained:

‘People in this community are enjoying circumcision; in my experience, what I expected from our children, though they are AmaMpondo, was that they show respect as shown by AmaXhosa who have been circumcised. These days they come back [from initiation school] drinking, smoking and with all the unexpected behaviour.’ (Elderly male, Headman 5, traditionally circumcised, IDI)

Similar views were shared by FGD participants who contended that this undesirable behaviour was learned during the initiation period, specifically attributing this new behaviour to negative teachings given to initiates by traditional nurses (*amakhankatha*). In this context, most men who had undergone MMC felt content with themselves and stated that they did not want their children to be circumcised traditionally, as they feared that their children would learn undesirable behaviour during the initiation period.

Moreover, the disrespect often shown by young circumcised men towards elderly men whom they view or know to be uncircumcised was also emphasised by another young man, in his own words:

‘One of the challenges facing circumcision is that other children don’t come back alive. Some change their behaviour and start smoking and drinking; there is no respect for their fathers and they call them boys.’ (Young male 2, medically circumcised, FGD 4)

#### Circumcision age eligibility

Data indicate that eligibility for circumcision in the study setting is determined by a number of factors, yet age appeared to be the key determining factor for eligibility to undergo TMC. This was not the case for the MMC method, as it is offered to males from the age of 16 for health reasons including cleanliness and protection from STIs (sexually transmitted infections) and HIV. Our data show that, in this setting, TMC is largely performed as a cultural rite of passage for boys who have reached the age of 18. In the interviews, a number of participants argued that age is important as a determining factor for eligibility to undergo TMC as it demonstrates a boy’s maturity and readiness for transition to manhood. Thus, this meant that boys who were medically circumcised below the age of 18 were not recognised as men by those who had undergone TMC. Thus age, as an eligibility criterion for undergoing circumcision, appeared to be a critical barrier to the integration of the two circumcision methods.

### Microsystem factors

#### The role of young men in alcohol and drug use

Interview data suggest that there is excessive use of drugs and alcohol during the initiation period, especially in the context of TMC. Several participants reported that initiates who undergo TMC get introduced to alcohol or other substances by traditional nurses (*amakhankatha*). One female traditional leader expressed that:

‘When these boys leave for the bush, we have hopes that we will achieve big things, but when they come back, it’s a great disappointment. They get teachings there such as smoking weed [marijuana], drinking alcohol and we would like to see a reduction in alcohol use as we really don’t know who is failing, either parents or traditional surgeons or nurses.’ (Middle-aged female, Traditional leader 1, IDI)

Male traditional nurses and circumcised young men were reported to be actively exerting pressure on initiates to use alcohol and drugs. This seems to be perpetuated by a dominant notion that drinking and smoking are essential markers of a transition from boyhood to manhood. This notion of manhood is emphasised in a narrative from a young circumcised man who explained that:

‘As part of the transition from boyhood to manhood, initiates should drink and smoke …’ (Young man 1, traditionally circumcised, IDI)

The participants further reported that the pressure to use alcohol and other drugs continued beyond the initiation period. Data suggest that this manner of demonstrating manhood forms part of a dominant narrative used to describe a ‘real man’ in this setting. According to the male youth culture in this setting, a circumcised man must demonstrate his manhood through engaging in activities like drinking alcohol. This was clearly illustrated in one of the FGDs, where an elderly woman described the behaviour of her sons who had been circumcised medically. She said:

‘Regardless of the method he has chosen, there is no difference in behaviour. I have sons who went to the hospital and they are not giving me support other than drinking alcohol and smoking marijuana and drugs, and I can’t lie, the situation is very bad there [initiation schools for MMC].’ (Elderly woman 1, FGD 3)

Other participants highlighted unrestricted access to initiation schools as a serious problem, resulting in the use of alcohol and drugs in the initiation lodges. Emphasis was made that this often occurs in initiation lodges of initiates who do not have adult male figures in their homes.

A young man posited:

‘If you don’t have an elderly brother or father, you end up being a victim.’ (IDI, traditionally circumcised young man 1)

#### The role of young men in the abuse of initiates

Some participants reported that abuse of initiates was not uncommon in this setting and contended that this was mainly because of unrestricted access to initiation schools. These participants reported that some traditional nurses and other young circumcised men deliberately inflict pain and torture initiates. While this was not a common viewpoint, some participants reported that abuse of initiates occurred irrespective of the circumcision method:

‘Sometimes there are deaths reported, cases of physical abuse and some end up in hospitals. Parents are not sure which method is safe because in TMC, they report deaths and in MMC, they get discriminated and physically abused [post-circumcision].’ (Young man 1, traditionally circumcised, IDI)

#### The role of parents, siblings and extended family members

Most participants reported that parents, siblings and family members play a crucial role in influencing young boys’ choice of their circumcision method. It was reported in both the FGDs and IDIs that when boys are ready for initiation, they often do not get an opportunity to choose whether to be circumcised traditionally or medically. Rather, senior family members make the decision on their behalf. The following narrative is explanatory:

‘If I have a child who is going for initiation, it’s me who takes the decision, together with my family male relatives.’ (Elderly man, Headman 2, traditionally circumcised, IDI)

Congruent with Headman 2, a male headman in one village said that:

‘A family has to go towards one direction to avoid conflicts, as we have witnessed different perceptions; for example, if they go to the mountain or hospital, they must all do that.’ (Elderly man, Headman 3, traditionally circumcised, IDI)

### Exosystem factors

#### Circumcision and financial gains

Data show that TMC is approached as a business by some people in this setting. In the interviews, a number of participants complained about the exorbitant amounts of money charged by traditional surgeons for performing the operation. Yet, data also show that young circumcised men find ways to exploit the custom for financial gain. This is indicated by the narrative given by a young man in an FGD who explained that young circumcised men actively recruit boys and convince them to choose their preferred method of circumcision so that they can gain financially. In his own words:

‘Sometimes when we have no boys willing to go for circumcision in that season, we embark on a door-to-door campaign to market our methods and traditional surgeons give us commission.’ (Young man 1, traditionally circumcised, FGD 2)

Linked to this, other participants talked about traditional nurses who charge the families of initiate’s exorbitant amounts of money, including being incentivised in other ways for looking after the initiates. The following extract illustrates this:

‘… this programme [TMC] is for financial gain; everyone claims to be a traditional nurse without a certificate.’ (Elderly man 2, FGD 1)

A number of participants stated that owing to the desire to make money, some traditional surgeons and nurses resist attempts to integrate the two circumcision methods, as they feel this could lead to them losing financially if the operation was done medically.

#### The role of community forums

In the context of MC, some participants described the role and impact of community forums on MC outcomes as critical. Accentuating the significance of community forums in curbing the abuse of initiates, one traditional surgeon opined:

‘The presence of local and district forums to monitor the situation has resulted in the reduction of physical abuse; there are no more beatings in the initiation schools.’ (Male, Traditional surgeon 2, IDI)

This view gained support from another traditional surgeon who similarly stressed the important role of community forums in the circumcision process. He posited:

‘Traditional leaders as the custodians of the tradition must use circumcision committees and community forums, as they are instrumental even during the signing of documents.’ (Male, Traditional surgeon 1, IDI)

### Macrosystem factors

#### Stigma and discrimination

Stigma and discrimination against medically circumcised men were reported to be common in this setting. In an FGD, a young man spoke about the discrimination suffered by medically circumcised men in his community, including social exclusion and ridicule. He explained that:

‘The disadvantage about being medically circumcised is that you are excluded in social activities.’ (Young man 2, medically circumcised, FGD 4)

Further highlighting the discrimination often suffered by medically circumcised men, another young man contributing in an FGD added that:

‘A real man is a man because of his manhood, and you have to show this, [but] in Pondoland we mislead each other and think that [only] if you are traditionally circumcised, you are a real man.’ (Young man 1, traditionally circumcised, FGD 4)

Data suggest that the stigmatisation and discrimination of medically circumcised men may have profound health and social effects on their lives. Some participants shared stories of medically circumcised men withdrawing from participating in social activities. These participants talked of medically circumcised men being gossiped about, ridiculed in public spaces and perceived as weak. This discrimination was reported to be more pronounced in social spaces such as schools and community events, as reported by a man in one of the FGDs:

‘… sometimes we get kicked out in some ceremonies and those from TMC stay inside …’ (Young man 4, medically circumcised, FGD 4)

Notwithstanding this, however, in the following extract, a 21-year-old man who had undergone TMC mentioned that even men who are traditionally circumcised do occasionally get discriminated against by medically circumcised men. He explained:

‘The problem of discrimination surfaces in community events such as graduation ceremonies, when we are drunk, where we call medically circumcised men imichweza [men who have had a smooth initiation period and healed fast due to the smaller size of their wounds] and they call us imigegemba [men who are homeless because we spend the initiation period in the bush or mountains].’ (Young man 1, traditionally circumcised, IDI)

While stigmatisation and discrimination against medically circumcised men was reported to be more common, there were some traditionally circumcised men in our sample who opposed the discrimination and stigmatisation of medically circumcised men. For example, a 42-year-old traditional surgeon gave his reasons for not discriminating against medically circumcised men:

‘I always meet people who have been medically circumcised and that’s not a problem because … it’s better to start in hospital then come back to get the teachings.’ (Male, Traditional surgeon 2, IDI)

#### Dominance of young men

In both the IDIs and FGDs, there was consensus among participants about youth dominance in the MC process. Most participants commented that young men dominated the MC process in their communities, with elderly men playing a very limited role in the process. The following extract is illustrative:

‘My opinion is that … when it’s initiation season, we should go out in numbers so that the youth can stop leading this custom.’ (Elderly woman 2, FGD 3)

Similarly, a number of elderly men participating in FGDs shared concerns that TMC was led by young men in their communities. Lamenting youth dominance in the custom, one elderly man posited:

‘If parents can reach an agreement, youth dominance in the circumcision process cannot be a problem … at the moment, the youth make their own decisions.’ (Elderly man 3, medically circumcised, FGD 1)

Data demonstrate that the young men’s dominance in the MC process benefits them, as they were reportedly able to make higher demands to the families of the initiates, as one young man explained:

‘We look at your financial capability and if your family can afford, we make sure that we ask for more money all the time …’ (Young man 1, traditionally circumcised, FGD 2)

## Discussion

The study aimed to explore factors influencing the integration of TMC and MMC at different socio-ecological levels and the findings show that the undesirable post-circumcision behaviour of newly circumcised men seeks to denigrate the significance of MC as a rite of passage to manhood. Scholars have similarly commented that initiation schools have historically been known to be key educational institutions for teaching positive and progressive societal norms and values.^19^ However, our findings confirm those of other studies conducted in the Eastern Cape province, which suggested that the role traditional circumcision schools once played in the socialisation of young AmaXhosa males has been eroded over the years,^20,21^ and that the teachings that are currently given to initiates, especially in TMC schools, are at odds with the dignity and self-respect expected from circumcised men.^22^ Integration of the two methods of male circumcision would be an opportunity to draw positive practices from both methods and find viable and acceptable ways of synergising such practices for the benefit of the young male population.

Previous research in this setting has shown that black communities are organised by age and gender, with the greatest authority vested in older men.^23^ Yet, the findings of this study show that young circumcised men reject the expectation to respect elderly men, especially those who are uncircumcised, thereby disrupting the social order in this setting. We argue that this may undermine the integration of the TMC and MMC methods, as some elderly participants doubted the value of TMC in producing respectful and productive men who can contribute in building their communities. As such, they were reluctant to allow their sons to be circumcised traditionally.

Findings reported in this article demonstrate that initiation schools are normally located in far-flung areas on the outskirts of the villages and highlight that this may be a key environmental factor that fuels the abuse of initiates, enabling it to occur unabated. This suggests the failure of the microsystem in providing a protective environment for initiates. As argued above, this is exacerbated by the fact that elderly men who are supposed to play a monitoring role were reportedly excluded from participating in the TMC processes, as they were perceived to be irrelevant by young circumcised men.

It has also been shown in this article that the pressure exerted on initiates to use alcohol and drugs is chiefly driven by their circumcised peers during and after circumcision. Some authors^2,3^ argue that traditionally circumcised men use alcohol as a way of demonstrating their manhood, to show that they are ‘real men’ compared to medically circumcised men. This comparison, and attachment of certain behaviours and practices to men, is partly based on their circumcision method and may lead to resistance towards the integration of TMC and MMC, because of the dominant perception that MMC is meant for men who are feeble and cannot withstand pain. This highlights the urgent need for focused male circumcision interventions targeted at young men in this setting, before and during the initiation period.^24^

This article presents findings that support Bronfenbrenner’s microsystemic influences on behaviour. This was done by showing that boys were likely to first hear about circumcision from, and be influenced to choose a method of circumcision by, their family members and friends. We thus argue that for the integration of MMC and TMC methods to be achieved in this setting, there is a need to explore and understand boys’ relationships with their primary social groups (family and peer networks) that provide not only social identity, support and role clarification, but also confusion. Social status by its very nature is believed to embrace fundamental pillars that are common to most African cultures, which include shared communal values, collectivism, coexistence and interdependence.^11,25^ This is because, as argued by other scholars,^15^ an increase in support by parents, siblings and peers may lead to stronger predictors of integration. However, like other studies,^26^ this study has shown that the majority of traditional leaders and community members in this setting promote TMC, and this is because of the perception that MMC and the inclusion of biomedical approaches to TMC threaten to erode the much valorised TMC as a cultural practice and rite of passage for black African males. This is worrying as emerging evidence suggests that an individual’s preference of circumcision method is based primarily on social considerations, rather than medical factors, especially in the Eastern Cape province.^27^

There is currently a strong call from the Eastern Cape traditional leaders for the community forums to be involved and to play a key role in the TMC processes. While this call is not based on empirical evidence, traditional leaders have argued that community forums are well placed to perform this role as they are conversant with, and trusted by, the communities. It is expected that their involvement in the TMC processes will significantly reduce complications related to TMC and curb deaths.^28^ Our findings support this view as many participants affirmed that community forums in this setting reduce abusive practices during initiation periods by forging constructive synergies between community members and male circumcision stakeholders. However, as argued by other authors,^26^ the resistance sometimes shown by traditional leadership towards the integration of TMC and MMC emanates partly from the fact that research findings and associated interventions have not been well presented and clearly communicated to them. The findings of this study are crucial in filling this gap. We argue that there is a need for a stronger collaboration between the South African Department of Health, the House of Traditional Leaders and other relevant stakeholders, with key targeted outcomes. This could include the promotion of research to inform strategies that can support community forums to perform their duties optimally.

Another key finding presented in this article was that some TMC practitioners (i.e. traditional surgeons, traditional nurses and young circumcised men) exploit the circumcision custom for financial gain. This finding reflects those of a recent study which showed that traditional surgeons in the Eastern Cape province were sometimes unqualified and unregistered young men who had been recently circumcised, but were already circumcising boys for economic gain.^28^ These findings are important as they add to the mounting body of literature that shows that TMC has been commercialised in the Eastern Cape province, with some people getting involved in the custom with the sole purpose of financial benefit.^29^

## Limitations

This was a qualitative explorative study to gain an in-depth understanding of the phenomena under study at the Ingquza Local Municipality, Eastern Cape. While the results of this study cannot be generalised, they may be transferred to a similar setting elsewhere. The data in this study were gathered through IDIs and FGDs, and this was done for the purpose of triangulation and to increase the trustworthiness of the study findings. Comparatively, similar questions were addressed in the FGDs and IDIs. IDIs enable the intricacies and contradictions of real lives to arise, without informants feeling obliged to construct public narratives or identities, while FGDs allow communally held thoughts and understandings of important issues to emerge, called a public account.^30^ In this study, IDIs and FGDs were employed to obtain individual and community insights. However, it is worth noting that different interview guides were used for each category of informant as opposed to the use of one interview guide with the same set of questions for all informants, and this is a possible limitation of this study. The interview guides encompassed somewhat similar questions for all informants, yet had specific questions related to each category of informant. Future studies should do well to use a single interview guide with the same set of questions for all categories of informant.

## Implications for policy and programmes

South Africa has a number of policies and statutes that, directly or indirectly, regulate male circumcision. These include the Constitution of the Republic of South Africa of 1996, HIV Testing Services Policy Guideline of 2016, *Children’s Act No. 38 of 2005* and *Application of Health Standards in Traditional Circumcision Act No. 6 of 2001*. The latter has been disowned by the traditional leaders, who claim that, as the rightful custodians of the cultural practice, they were never consulted in the development of the policy. While these policies and statutes have contributed to improving male circumcision outcomes in the Eastern Cape province, deaths and poor health outcomes of initiates continue to be reported, highlighting that so far the attempts to regulate male circumcision have had limited success.^2,29^

In line with this, and as has been noted by other authors, collaborative efforts between stakeholders such as the Department of Health, traditional leaders and the local communities are urgently needed to address challenges related to male circumcision in the Eastern Cape province. In addition, evidence-based health-promotion programmes that will increase awareness about the importance of health screening of boys prior to TMC are needed.

Our findings have shown numerous factors affecting TMC, including poor management of initiation schools, physical abuse of initiates and lack of discipline and respect following MC. The current policies in both TMC and MMC need to be reviewed and amended to stipulate procedures of operations and management. Harmful practices occurring during the circumcision period need to be outlawed and made punishable by law.^29^ In the past decade, TMC was only the responsibility of the traditional leadership; however, integrated efforts are required to curb the reported adverse events. There appears to be huge competition between the two methods which initiates choose. Better strategies to reduce this conflict between the two methods should be developed.

## Conclusion

This article contributes to existing literature on male circumcision as it broadly explored the socio-ecological factors influencing the integration of TMC and MMC methods in rural South Africa. In this study, we demonstrated how these factors, which operate at individual, social and structural levels, interface to influence the integration of TMC and MMC in the Ingquza Hill Local Municipality. Based on the findings of this study, we argue that it is essential to consider an integrated approach that will address the individual and societal concerns about MMC, including positive practices (e.g. sterilisation of materials used in the initiation schools and use of gloves) and integrating them into TMC to make it safer and more broadly acceptable. However, more research is needed to deepen the understanding of how the integration of TMC and MMC can be implemented successfully in traditionally circumcising communities in South Africa.
